# The Role of Cardiac Magnetic Resonance in Evaluation of Idiopathic Ventricular Arrhythmia in Children

**DOI:** 10.3390/jcm10071335

**Published:** 2021-03-24

**Authors:** Halszka Kamińska, Łukasz A. Małek, Marzena Barczuk-Falęcka, Marta Bartoszek, Ewa Strzałkowska-Kominiak, Mikołaj Marszałek, Ewa Brzezik, Michał Brzewski, Bożena Werner

**Affiliations:** 1 Department of Pediatric Cardiology and General Pediatrics, Medical University of Warsaw, 02-091 Warsaw, Poland; halszka.kaminska@wum.edu.pl (H.K.); bozena.werner@wum.edu.pl (B.W.); 2 Department of Epidemiology, Cardiovascular Disease Prevention and Health Promotion, National Institute of Cardiology, 04-635 Warsaw, Poland; 3 Department of Pediatric Radiology, Medical University of Warsaw, 02-091 Warsaw, Poland; marz.barczuk@gmail.com (M.B.-F.); drmartabartoszek@gmail.com (M.B.); ewa.brzezik@uckwum.pl (E.B.); mbrzewski@me.com (M.B.); 4 Faculty of Mathematics and Information Science, Warsaw University of Technology, 00-662 Warsaw, Poland; ewa.kominiak@gmail.com; 5 English Division, Medical University of Warsaw, 02-109 Warsaw, Poland; s075803@student.wum.edu.pl

**Keywords:** cardiac magnetic resonance, late gadolinium enhancement, idiopathic ventricular arrhythmia, children

## Abstract

The aim of the study was to assess the role of cardiovascular magnetic resonance (CMR) in the diagnosis of idiopathic VA in children. This retrospective single-centre study included a total of 80 patients with idiopathic ventricular arrhythmia that underwent routine CMR imaging between 2016 and 2020 at our institution. All patients underwent a 3.0 T scan involving balanced steady-state free precession cine images as well as dark-blood T2W images and assessment of late gadolinium enhancement (LGE). In 26% of patients (*n* = 21) CMR revealed cardiac abnormalities, in 20% (*n* = 16) not suspected on prior echocardiography. The main findings included: non-ischemic ventricular scars (*n* = 8), arrhythmogenic right ventricular cardiomyopathy (*n* = 6), left ventricular clefts (*n* = 4) and active myocarditis (*n* = 3). LGE was present in 57% of patients with abnormal findings. Univariate predictors of abnormal CMR result included abnormalities in echocardiography and severe VA (combination of >10% of 24 h VA burden and/or presence of ventricular tachycardia and/or polymorphic VA). CMR provides valuable clinical information in many cases of idiopathic ventricular arrhythmia in children, mainly due to its advanced tissue characterization capabilities and potential to assess the right ventricle.

## 1. Introduction

Ventricular arrhythmia (VA) is a prominent problem in the field of paediatric cardiology. In contrast to the adult population, it mostly affects patients with no apparent cardiovascular pathology and as such is considered to be idiopathic, meaning of unknown origin [1]. The most common forms of VA in children are premature ventricular complexes (PVCs), usually originating from the right or left ventricular outflow tract. They often require detailed diagnostic analysis in the search of underlying and potentially dangerous pathology [2,3,4,5]. Identifying the substrate of arrhythmia has been a goal of many studies published throughout the course of recent years, many of which promote the role of CMR in this diagnostic process, particularly due to its capability to localize the source of electrical instability as well as a mean of guiding the electrophysiological treatment [6,7,8]. All of these studies were conducted on adult subjects and addressed mainly late gadolinium enhancement (LGE) and its role in tissue characterization in the search for aetiology of myocardial injury [9,10]. The recent study published by Andreini et al. documented that one fourth of adult patients with ventricular arrhythmia and no abnormalities in echocardiogram had abnormal CMR results [8]. Still, little is known about the diagnostic value of CMR in children with arrhythmia. Therefore, the aim of our study was to assess the role of CMR in identifying underlying structural heart disease and its role as a potential substrate of VA considered as idiopathic in paediatric population.

## 2. Materials and Methods

### 2.1. Study Group

Our single-centre study was a retrospective analysis of 80 consecutive children suffering from supposedly idiopathic VA in whom CMR was performed between April 2016 and January 2020 due to clinical indications described below. These patients belong to a group of a total of 390 CMR scans performed during that period (20.5% of all the referrals).

Only children in whom initial diagnostic work-up failed to identify arrhythmic substrate and therefore still considered idiopathic while referred for MRI were included into the study group.

### 2.2. Pre-CMR Evaluation

In all patients, the type of arrhythmia was assessed in ECG and quantified in a 24-h Holter ECG-monitoring. Morphology of PVCs (left or right bundle branch block, or other; polymorphic or monomorphic type), 24 h burden of arrhythmia (<10%, 10–20%, >20%) and complexity of VA (presence of sustained or non-sustained ventricular tachycardia—VT) were determined. The distinction between severe and non-severe form of the disease was defined as a numerous extrasystole (>10% PVCs/24 h) or a complex form of VA (ventricular tachycardia, polymorphic arrhythmia) versus <10% PVCs/24 h without VT and the lack of complex VA forms.

All children underwent an echocardiographic evaluation (Philips, Cambridge MA, USA; Epiq 7 system with S5-2/S8-3 transducer) performed by an experienced sonographer for a baseline two-dimensional assessment of cardiac anatomy, and chamber’s size and function; all in accordance to standard clinical recommendations and a set of norms established for the paediatric population [11,12].

CMR was the next step of diagnostic work-up in patients with baseline echocardiographic parameters conforming to normal limits, but with coexisting markers of severe arrhythmia or echocardiogram’s results aberrant, but not specific for any disease or lacking diagnostic criterion’s strength. CMR was performed during the same hospital stay. Patients with a clear diagnosis after echocardiography that were referred for CMR as a means of further assessment were excluded from the analysis.

### 2.3. CMR Imaging and Assessment

Imaging was performed with a Siemens MagnetomSkyra 3 Tesla scanner (Siemens, Erlangen, Germany). The protocol included cine steady-state free precession (SSFP) breath-hold sequences in two-, three-, four-chamber long-axis views, and short-axis views covering the ventricles from the mitral and tricuspid annular plane to the apex. In patients with suspected ARVC an axial cine stack was also obtained. Imaging parameters were as follows: field of view 340 mm, matrix 208, repetition time approximately 39.24 ms, echo time 1.43 ms, flip angle 39 degrees, slice thickness 6–8 mm (depending on the child age), gap 1–2 mm, in-plane image resolution 1.6 × 1.6 × 6–8 mm, temporal resolution 25 phases per cardiac cycle. This was followed by acquisition of dark-blood T2W images with fat suppression in two, three- and four-chamber views and three short-axis slices (basal, mid-ventricular and apical) for evaluation of myocardial edema. Finally, 0.1 mmol/kg of a gadolinium-based contrast agent (gadobutrol—Gadovist^®^, Bayer Pharma AG, Berlin, Germany), flushed with 20–30 mL of isotonic saline was administered intravenously. Imaging parameters were as follows: Field of view 340 mm, matrix 256, repetition time approximately 1600–1800 ms, echo time 44 ms, flip angle 180 degrees, slice thickness 6–8 mm (depending on the child age), in-plane image resolution 1.6 × 1.6 × 6–8 mm. LGE images in three long-axis planes and a short-axis stack were obtained with a breath-hold phase-sensitive inversion recovery sequence (PSIR) 10–15 min after contrast injection. The inversion time was set with means of the initial TI scout to completely null normal myocardium (typically between 250 and 350 ms).

Images were analysed using validated software—MRHeart plugin for Horos [13]. On cine images, epi- and endocardial contours in end-diastole and endocardial contours in end-systole were manually traced on the short-axis views. Delineated contours were used for the quantification of left ventricular (LV) and right ventricular (RV) volumes, ejection fraction and mass. Volumes and masses were then indexed for body surface area and compared to published normal values [14]. Myocardial oedema was assessed by comparison of signal intensity of the myocardium regionally and with skeletal muscle on T2-weighted images [15,16]. The presence of LGE and left ventricular clefts was assessed visually. All assessments were performed by two readers experienced in CMR (cardiologist with 12 years of expertise and radiologist with four years of expertise).

Acute myocarditis was diagnosed according to the updated Lake Louise criteria using a T2-based criterion with a T1-based criterion [16]. In order to be diagnose acute myocarditis patients had to have signs of myocardial oedema and a predominantly non-ischemic LGE pattern.

The presence of CMR criteria for arrhythmogenic right ventricular cardiomyopathy (ARVC) was considered according to 2010 Task Force document [17].

### 2.4. Study Endpoint

For the study endpoint we adopted the abnormal result of CMR considered as a potential arrhythmogenic substrate, including: isolated myocardial scarring, presence of diagnostic criteria for ARVC, left ventricular clefts and active myocarditis.

### 2.5. Post CMR Follow-Up

All the patients were followed-up consecutively in accordance to the severity of the disease. Information about the clinical course and therapy was collected from the hospital documentation.

### 2.6. Ethical Considerations

The study was approved by the Medical University of Warsaw Ethics Committee (no. KB/13/2017 issued on 2 February 2017) and was conducted in accordance with the Declaration of Helsinki. In all patients, written informed consent was obtained from parents and children older than 16 years of age.

### 2.7. Statistical Methods

Categorical variables were presented as counts and percentages, while continuous variables were shown as mean and standard deviation (SD). Fisher’s exact test was used to compare categorical variables. Student’s *t* test was applied to compare continuous variables. The inter-observer and intra-observer variability for the continuous MRI variables were tested in 10 randomly selected patients using Pearson correlation coefficient and interclass correlation coefficient (ICC). For CMR categorical variables, a Cohen kappa test was performed. All tests were two-sided with the significance level set at *p* < 0.05. Statistical analyses were performed with R open-source software.

## 3. Results

### 3.1. Baseline Characteristics

The summary of patient’s characteristics at the time of qualification for CMR is presented in Table 1.

Almost all patients were asymptomatic. To the exceptions belonged three children diagnosed later with active myocarditis, of whom two suffered from chest pains, and the youngest patient (2.8-years old girl) presented heart failure symptoms evolving into cardiogenic shock secondary to sustained polymorphic ventricular tachycardia. Other four children with excessive ventricular extrasystole reported palpitations and two poor exercise tolerance. Twenty-four children (with severe or symptomatic arrhythmias) received pharmacological treatment (β-blocker, amiodarone, propaphenone or flecainide). No arrhythmic syncopes or sudden cardiac deaths were recorded in our studied group prior to MRI.

### 3.2. CMR Findings

In 16 children (20%) PVCs produced artifacts, without significant influence on the quality of CMR images (moderate to good quality). In another 8 children (10%) the quality of MRI due to artifacts was poor making the detailed assessment of ventricular function inadequate, but permitting LGE assessment.

**Figure 1:**
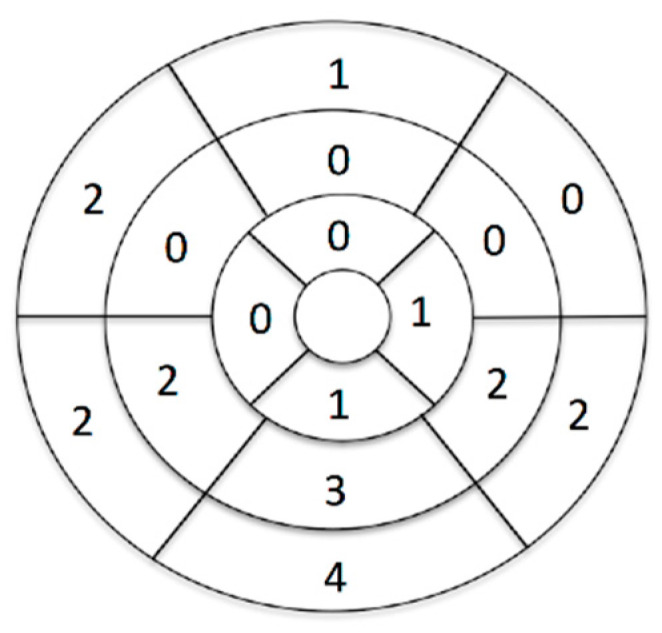
Number of patients with LGE in each of the left ventricular segments.

CMR results showed abnormalities interpreted as potential arrhythmic substrates in 21 patients (26%). In 10 children these were anatomical/functional anomalies including enlarged/dyssynchronic ventricles or single/multiple ventricular clefts and in 12 patients abnormal late gadolinium enhancement (LGE) was recorded. It was present mainly in the left ventricle (11 patients, 92%) and allowed for diagnosis of active myocarditis (if accompanied by myocardial oedema) or non-ischemic (mid-wall/subepicardial) type of isolated fibrosis (in the absence of myocardial oedema). Localization of the LGE in the left ventricle according to a 17-segment model is presented in Figure 1. In one patient with ARVC LGE was a marker of right ventricular fibrosis.

In 16 patients (20%) the abnormal CMR results were new findings, not suspected after prior echocardiography. In the remaining 5 children two-dimensional echocardiography showed abnormalities suggestive of ARVC, but lacking the strength of criterion: in 3 children RV outflow tract dilatation with preserved global and regional systolic function and in other 2 children isolated RV free wall dyssynchrony. In all five aforementioned patients CMR provided additional information allowing for fulfilment of imaging diagnostic criteria (additional minor criterion in 4 children and major in one patient): in 3 patients with dilated RV outflow tract, the indexed RV end-diastolic volume was 92 mL/m^2^ (in two girls) and 112 mL/m^2^ in one boy (fulfilling major criterion) with visible regions of RV dyskinesia. Regional RV wall dyssynchrony present in two patients on echocardiography was confirmed in CMR and accompanied by small volumetric criterion in 1 case (indexed RV end-diastolic volume 93 mL/m^2^ in a girl) or globally deteriorated systolic function (RVEF = 42%) and right ventricular fibrosis in one patient. Among the patients with the presence of ARVC criteria in CMR (*n* = 6), echocardiography failed to detect RV abnormalities in one child. In all children fulfilment of additional CMR diagnostic criteria allowed to establish the diagnosis of ARVC.

Among four children diagnosed with LV clefts, in two the myocardial wall recesses were multiple and in two, single, but with an uneven and irregular structure of the surrounding muscle.

In 59 children (74%) CMR showed normal cardiac morphology and no LGE.

**Figure 2:**
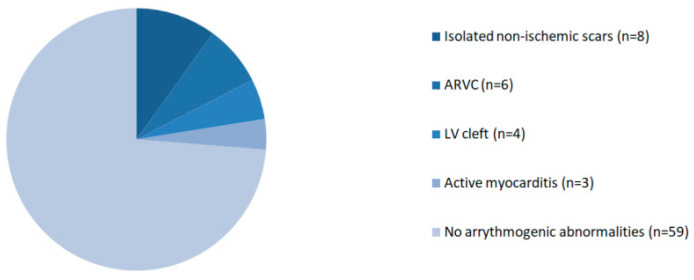
Abnormal CMR findings. ARVC—arrhythmogenic right ventricular cardiomyopathy.

The CMR findings considered as potential sources of arrhythmia are reported in Figure 2.

**Figure 3:**
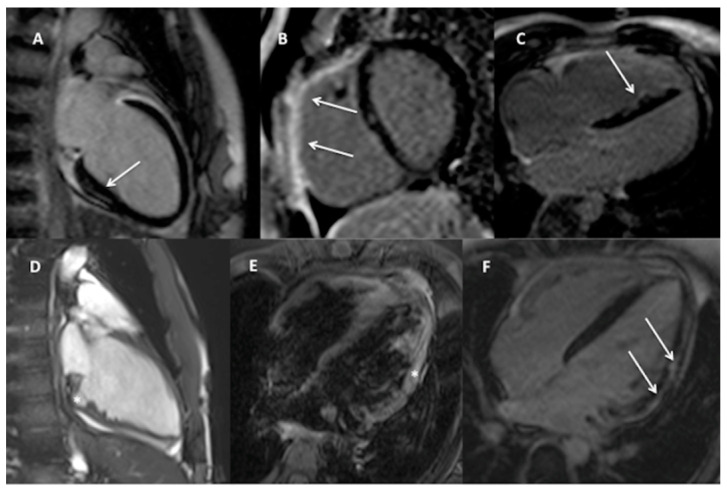
Examples of potentially arrhythmogenic CMR findings in the studied cohort: (**A**). Two-chamber view, non-ischemic, subepicardial area of fibrosis in the basal infero-septal segment of the LV (arrow) without signs of myocardial oedema (not shown) in a 16-year-old girl with 12%/24 h of complex LBBB-like PVCs suggestive of previous myocarditis; Band (**C**)—regions of LGE (arrows) in the RV outflow tract and free wall ((**B**)—short-axis view) and in the interventricular septum (**C**)—4-chamber view) in a 17-year old boy with <1%/24 h complex LBBB-like PVCs and an overall small MRI criteria for ARVC. (**D**). Two-chamber cine view in end-diastole showing large crypt (asterisk) and irregular myocardial pattern of the inferior LV wall in a 13-year-old boy with 14%/24 h of RBBB-like PVCs, (**E**,**F**). 4-chamber view, signs of myocardial oedema (E—asterisk) and subepicardial, non-ischemic stria of fibrosis (F—arrows) in the lateral LV wall in a 14-year-old boy with <1%/24 h of complex undetermined PVCs indicative of acute myocarditis.

The examples of detected findings are shown on Figure 3.

The distribution of patients’ clinical features and CMR volumetric and functional analysis in relation to study endpoints are summarized in Table 2 and Table 3.

Analysis of the prevalence of clinical features and their distribution within the group of patients with and without abnormal CMR results showed significant difference in number of PVCs on 24-h Holter-ECG monitoring. Abnormal CMR findings were more frequent in children with 10–20%/24 h PVCs and less frequent in children with <10%/24 h PVCs than normal CMR result (*p* = 0.01 and *p* = 0.02 respectively). Interestingly, the same ratio of patients with more than 20% of arrhythmia in 24 h was seen in both groups (*p* = 1.0). Age, BSA, sex, sports participation and clinical symptoms were not significantly different between the groups, as were not the incidence of complex or polymorphic forms of arrhythmia.

Also in the volumetric and functional cardiac assessment no statistically significant difference was noted between the groups with and without potentially arrhythmogenic abnormalities discovered in CMR.

The distribution of the abnormalities reported in CMR and interpreted as arrhythmogenic substrates within the examined population in relation to the severity of arrhythmia is presented in Table 4.

Among the total number of 21 abnormal CMR findings all but two were detected in subgroup with severe arrhythmia (*p* = 0.014). Only one patient with ARVC and one child with isolated left ventricular fibrosis (non-ischemic scars) had <10% PVCs over 24 h. Similar proportion was observed in LGE distribution: Eleven cases in the subgroup with severe arrhythmia and only one in the group with a benign form of the disease. All the cases with active myocarditis and left ventricular clefts were found in patients with severe arrhythmia.

### 3.3. Reproducibility Analysis

Very low inter- and intra-observer variability were noted for analyzed CMR measurements including LVEDV, LVEF, LV mass, RVEDV and RVEF. Correlation coefficients for inter-observer variability ranged from 0.99 for LVEDV to 0.96 for RVEF, all *p* < 0.001. Correlation coefficients for intra-observer variability ranged from 0.97 for LVEDV to 0.94 for LV mass, all *p* < 0.001.ICC ranged between 0.96 and 0.98 for inter-observer variability and 0.98–0.99 for intra-observer variability. The Cohen kappa values for the inter-observer and intra-observer variability of categorical CMR parameters were 0.91 and 0.97 for LGE and 0.88 and 0.94 for LV oedema detection.

### 3.4. Follow-Up

All the children were followed-up after CMR for a mean of 20 ± 5 months and the data were available for interpretation. No sudden cardiac incidents including ventricular fibrillation, successfully defibrillated or leading to death were registered within studied population. No ICD implantation was required. In all three children with active myocarditis the arrhythmia dissolved after completion of the healing process and no residual myocardial fibrosis was observed in control CMR after 6–8 months as analysed in another study from our research group [18]. Electrophysiological study and ablation were performed in five patients: Four of them had no pathological findings in CMR and in one CMR was helpful in establishing the diagnosis of ARVC and pointing the potential to region of interest. In children with normal cardiac morphology the ablation was performed either due to excessive or symptomatic character of the arrhythmia or patients’ willingness to participate in competitive sports. The single patient with ARVC and numerous PVCs was qualified for ablation to reduce arrhythmic burden.

## 4. Discussion

Our study shows that even in children with arrhythmia considered as idiopathic it is worth searching for underlying structural heart disease and that CMR may be a valuable tool for this task. Children with benign forms of VA, no symptoms, no high-risk points in family history and no abnormalities in echocardiography do not typically require diagnostic escalation. Patients with severe arrhythmia were the most numerous group among the patients requiring CMR and they had presented higher incidence of pathological findings than subjects with less severe forms of the disease, although the statistically significant disproportion between subgroups was recorded only for the combined endpoint, which may reflect the small number of the total population. Three-fourths of our studied group remained in the idiopathic arrhythmias’ pool after CMR assessment, including almost two thirds of patients with severe forms of the disease.

The abnormalities considered as potentially arrhythmogenic were detected by CMR in one fourth of our patients and in one fifth they were new findings, not registered by previous echocardiographic evaluation. Considering high occurrence of arrhythmias labelled as idiopathic in paediatric population, this number is surprisingly high. However, it is consistent with the results published recently by Andreini et al. and derived from the adult population suffering from ventricular arrhythmia with no abnormalities described in echocardiography [8].

The possible arrhythmogenic substrates identified in our population by CMR were different and fewer than those described in adults [8]. This was related to the fact that patients with a clear diagnosis on prior echocardiography and, therefore, non-idiopathic forms of VA were excluded from our analysis. Many of the CMR discoveries were based on the presence of LGE allowing for diagnosis of myocarditis or isolated fibrosis of myocardial tissue (scars). In our population active myocarditis was the least common arrhythmogenic pathology (diagnosed only in 3 children), although with an undisputed link to VA. In all those patients the resolution of the inflammatory process was related to a gradual termination of arrhythmia. Patients with isolated LGE and no oedema who did not fulfil the criteria for diagnosis of active myocarditis represented the largest group of abnormal CMR results. In contrast to the adult population the character of fibrosis was non-ischemic in all children, possibly a remnant of a prior inflammation or due to developing cardiomyopathy. The role of this finding is not easy to establish, especially with no literature on the subject based on paediatric population. The recent paper by Shanbhag et al. analysed 900 older adult patients in whom LGE was assessed in CMR and classified into ischemic and non-ischemic pattern for potential cardiovascular risk stratification [19]. In our population all cases of fibrosis represented mid-wall type and were located in regions typical for myocarditis, with no additional left ventricular enlargement or deteriorated systolic function. Those features pointed to post-inflammatory character of fibrosis rather than developing cardiomyopathy. No residual fibrosis on repeated CMR was detected in 3 children with CMR-based diagnosis of active myocarditis in whom arrhythmia had resolved [19].

LGE was observed in only one among 6 children with ARVC. Fibrosis revealed in CMR is not currently included into revised Task Force Criteria, even if fibro-fatty replacement of myocardial free wall tissue is considered as a major criterion in myocardial biopsy [17,20]. LGE marked fibrosis is definitely more often seen in adults with ARVC, in whom the disease lasts longer and gradual myocardial replacement is more advanced than in children [21,22]. However, it is the follow-up of children with ARVC and no fibrosis that may be helpful in offering additional data on relation between fibrosis progression and the evolution of arrhythmia in affected individuals. The problem certainly calls for further studies.

In 4 children with excessive arrhythmia CMR provided a diagnosis of left ventricular clefts, which are easy to overlook in echocardiography. A cleft is defined as small muscular recess with preserved surrounding muscle structure and contractility, with no specific location and generally asymptomatic [23]. On the other hand, among all the “outpouchings” of the ventricular wall, clefts have the highest incidence of associated anomalies. Especially hypertrophic cardiomyopathy (HCM) is noted as often coexisting or even being predated by diagnosis of multiple, but not single clefts, for which CMR is known to be the most potent modality [24]. In two of our patients the clefts were single, however, the surrounding muscle was uneven and irregular, which may be a stage preceding further clefts evolution. Small retrospective studies and case reports advocate relation between left ventricular clefts evolving into non-compaction (NC) phenotype, but mostly in adults [25,26,27]. Still, it is difficult to exclude clefts being possible early cardiomyopathic manifestations in children as potential arrhythmogenic substrates.

Analysing CMR results and detected abnormalities we included left ventricular clefts and non-ischemic LV scars into the potential arrhytmogenic substrates’ pool. However, it is hard to unequivocally assess their actual relevance in triggering of ectopy. While literature is adamant on the arrhythmogenic role of fibrosis in adults and the prognostic role of multiple clefts in the foretelling of HCM, their significance is not thoroughly researched in children. However, recognition of these abnormalities in patients in whom ventricular arrhythmia is considered to be idiopathic may be of importance for the future arrhythmic assessment within the paediatric population.

The temptation to include the CMR as a standard step in paediatric ventricular arrhythmias’ diagnostic work-up is great indeed. If we note that a quarter of patients considered as idiopathic VA turn up to have abnormal CMR results, we have to ask ourselves: should we settle on echocardiogram for all the answers? Particularly in the case of non-ischemic myocardial scars and ventricular clefts the long follow up will be crucial for the assessment of their role in arrhythmogenesis and potential indications for treatment. As our results show, the pool of “idiopathic ventricular arrhythmias” in children may not be as extensive as originally believed.

### Study Limitations

Some limitations of our study should be listed. Small cohort size could undermine the credibility of the statistical analysis, especially of the smaller samples within the studied group. A single centre engaged in the process may not be an accurate representation of the whole population. Also, as any retrospective study the analysis is limited due to the absence of a prospective design and follow-up. This being an original paper, the significance of detected abnormalities recognized as responsible for potential arrhythmogenesis, especially isolated left ventricular fibrosis and left ventricular clefts, is treated as given for adults only. We expect this to extrapolate to the paediatric population, albeit to an as-of-yet unknown degree of certainty. None of our findings from that group has been verified in invasive electrophysiology study and/or electro-mapping. Finally, this study was performed at a time when the parametric T1/T2 mapping techniques were unavailable in our centre, which may have been able to detect more subtle forms of myocardial injury [28]. More research with increased sample sizes is needed.

## 5. Conclusions

CMR provides valuable clinical information in many cases of idiopathic ventricular arrhythmia in children, mainly due to its advanced tissue characterization capabilities and potential to assess the right ventricle.

## Figures and Tables

**Table 1:** Patients’ baseline characteristics.

Feature	Study Group *n* = 80
Mean age, SD [years]	13.1 ± 3.6
Male sex	48 (60%)
PVCs morphology	LBBB 65 (81%) RBBB 10 (12.5%) Undetermined 5 (6.5%)
>10% PVCs/24 h	48 (60%)
Complex PVCs	27 (34%)
Polymorphic PVCs	13 (16%)
**Severe VA** (>10%PVCs/24 h *OR* complex forms)	55 (69%)
**Symptoms**	9 (11%)
Palpitations	4
Poor exercise tolerance	2
Chest pain	2
Cardiogenic shock	1
**Pharmacotherapy**	24 (30%)
Metoprolol	12
Sotalol	5
Amiodarone	4
Flecainide + metoprolol	4
Propaphenone	2

PVCs—premature ventricular contractions, VA—ventricular arrhythmia.

**Table 2:** Clinical characteristics of patients in relation to study endpoints.

Clinical Characteristic		Abnormal CMR Result (*n* = 21)	Normal CMR Result (*n* = 59)	*p*-Value
Mean age (years)		14.4	13.2	0.165
BSA		1.58 ± 0.23	1.49 ± 0.33	0.215
Male sex		12 (57%)	36 (61%)	0.799
Sport participant		3 (14%)	8 (14%)	1
Symptoms		4 (19%)	5 (8%)	0.232
Abnormal echocardiography		5 (23%)	0 (0%)	0.001
Arrhythmia				
Total PVCs burden	PVCs < 10%/24 h	4 (19%)	29 (49%)	0.020
	PVCs 10–20%/24 h	9 (43%)	8 (14%)	0.011
	PVCs > 20%/24 h	8 (38%)	22 (37%)	1
Complex forms (nsVT or VT)		10 (48%)	18 (30%)	0.188
Polymorphic arrhythmia		4 (19%)	9 (15%)	0.735
Pharmacotherapy		8 (38%)	16 (27%)	0.409

BSA—body surface area, nsVT—non-sustained ventricular tachycardia, PVC—premature ventricular complexes, VT—ventricular tachycardia.

**Table 3:** CMR measurements in association with study endpoints.

CMR Results	All Patients (*n* = 80)	Abnormal CMR Result (*n* = 21)	Normal CMR Result (*n* = 59)	*p*-Value
LVEDVi [mL/m^2^]	84.85 ± 26.59	83.40 ± 20.35	85.34 ± 28.57	0.743
LVESVi [mL/m^2^]	34.21 ± 24.27	34.20 ± 9.51	34.21 ± 26.86	0.998
LVSVi [mL/m^2^]	50.59 ± 8.31	49.75 ± 9.51	50.89 ± 7.92	0.634
LVEF [%]	61.28 ± 7.64	60.10 ± 7.99	61.71 ± 7.54	0.440
LV mass index [mL/m^2^]	57.68 ± 12.72	58.05 ± 13.86	57.55 ± 12.43	0.889
RVEDVi [mL/m^2^]	89.39 ± 15.77	87.65 ± 12.39	89.96 ± 16.79	0.547
RVESVi [mL/m^2^]	38.30 ± 10.54	37.35 ± 9.01	38.62 ± 11.07	0.641
RVSVi [mL/m^2^]	51.13 ± 9.32	50.00 ± 8.88	51.52 ± 9.52	0.554
RVEF [%]	57.54 ± 6.13	57.47 ± 6.98	57.56 ± 5.89	0.963
LGE	12	12	0	<0.0001

LVEDVi—left ventricular end-diastolic volume index, LVESVi—left ventricular end-systolic volume index, LVSVi—left ventricular systolic volume index, LVEF—left ventricular ejection fraction, RVEDVi—right ventricular end-diastolic volume index, RVESVi—right ventricular end-systolic volume index, RVSVi—right ventricular systolic volume index, RVEF—right ventricular ejection fraction, LGE—late gadolinium enhancement; *p*-value ilustrates the comparison between the group with normal and abnormal CMR result.

**Table 4:** Potential substrates of arrhythmia in children with ventricular ectopic beats in relation to the severity of arrhythmia.

CMR Result	<10% PVCs/24 h and Nocomplex Forms (*n* = 25)	>10% PVCs/24 h *OR* Complex Forms (*n* = 55)	*p*-Value
Abnormal CMR findings	2 (8%)	19 (35%)	0.014
Detailed analysis			
All cases with LGE	1 (4%)	11 (20%)	0.092
Isolated non-ischemic scar	1 (4%)	7 (13%)	0.424
Criteria for ARVC	1 (4%)	5 (9%)	0.660
LV clefts	0 (0%)	4 (7%)	0.304
Active myocarditis	0 (0%)	3 (5%)	0.548

LV—left ventricle, PVCs—premature ventricular complexes, VT—ventricular tachycardia.

## Data Availability

The data presented in this study are available on request from the corresponding author. The data are not publicly available due to privacy.
